# Phylogeny of the Vitamin K 2,3-Epoxide Reductase (VKOR) Family and Evolutionary Relationship to the Disulfide Bond Formation Protein B (DsbB) Family

**DOI:** 10.3390/nu7085281

**Published:** 2015-07-29

**Authors:** Carville G. Bevans, Christoph Krettler, Christoph Reinhart, Matthias Watzka, Johannes Oldenburg

**Affiliations:** 1 Im Hermeshain 6, 60388 Frankfurt am Main, Germany; E-Mail: bevans@jhu.edu; 2 Department of Molecular Membrane Biology, Max Planck Institute of Biophysics, 60388 Frankfurt am Main, Germany; E-Mails: christoph.krettler@biophys.mpg.de (C.K.); christoph.reinhart@biophys.mpg.de (C.R.); 3 Institute of Experimental Haematology and Transfusion Medicine, University Clinic Bonn, 53105 Bonn, Germany; E-Mail: matthias.watzka@ukb.uni-bonn.de

**Keywords:** cyclic permutation, DsbB, homology modeling, phylogeny, sequence conservation, vitamin K, vitamin K 2,3-epoxide, VKOR, VKORC1, VKORC1L1

## Abstract

In humans and other vertebrate animals, vitamin K 2,3-epoxide reductase (VKOR) family enzymes are the gatekeepers between nutritionally acquired K vitamins and the vitamin K cycle responsible for posttranslational modifications that confer biological activity upon vitamin K-dependent proteins with crucial roles in hemostasis, bone development and homeostasis, hormonal carbohydrate regulation and fertility. We report a phylogenetic analysis of the VKOR family that identifies five major clades. Combined phylogenetic and site-specific conservation analyses point to clade-specific similarities and differences in structure and function. We discovered a single-site determinant uniquely identifying VKOR homologs belonging to human pathogenic, obligate intracellular prokaryotes and protists. Building on previous work by Sevier *et al.* (*Protein Science* 14:1630), we analyzed structural data from both VKOR and prokaryotic disulfide bond formation protein B (DsbB) families and hypothesize an ancient evolutionary relationship between the two families where one family arose from the other through a gene duplication/deletion event. This has resulted in circular permutation of primary sequence threading through the four-helical bundle protein folds of both families. This is the first report of circular permutation relating distant α-helical membrane protein sequences and folds. In conclusion, we suggest a chronology for the evolution of the five extant VKOR clades.

## 1. Introduction

In humans and other animals, vitamin K 2,3-epoxide complex subunit 1 (VKORC1) is the primary oxidoreductase enzyme that reduces vitamin K quinone (K), acquired in trace amounts by dietary uptake, to the hydroquinone (KH_2_) form, functioning as the point of entry for vitamin K into the vitamin K cycle [1,2,3]. Subsequently, vitamin K hydroquinone is oxidized to vitamin K 2,3-epoxide (K>O) during the posttranslational activation of vitamin K-dependent (VKD) proteins involving enzymatic conversion of specific glutamate (Glu) residues to γ-carboxyglutamate (Gla) residues. To complete the cycle, VKORC1 sequentially reduces K>O to K and KH_2_, ensuring that the limiting trace amounts of vitamin K are efficiently recycled to drive further rounds of γ-glutamyl carboxylation. VKORC1 has been demonstrated to be essential to the production of VKD blood clotting factors which takes place in liver hepatocytes [4]. Suboptimal availability of K from dietary sources can eventually result in interruption of vitamin K cycle turnover and lead to pathophysiological anticoagulation and, in the extreme case, uncontrollable bleeding [3,5]. In the case of pathological hypercoagulative conditions such as thrombosis and embolism, 4-hydroxycoumarin based oral anticoagulants, including warfarin as a well-known example, are administered to block the enzymatic function of VKORC1, effectively diminishing turnover of the vitamin K cycle [6]. This results in induction of a controlled hypocoagulative state to counteract the tendency towards clot formation. Thus, the biological action of vitamin K on hemostasis, mediated by function of the VKORC1 enzyme, is entirely dependent on vitamin K nutritional status and, occasionally, on treatment with oral anticoagulant drugs. Very recently, evidence has been presented that a second VKOR enzyme, VKORC1-like 1 (VKORC1L1), is responsible for extrahepatic VKOR activity crucial to bone growth and homeostasis [7,8]. Interestingly, although VKORC1L1 carries out the same two enzymatic steps of the vitamin K cycle as does VKORC1, it appears to be far less sensitive to oral anticoagulant drugs, suggesting that its biological activity may not be substantially reduced when oral anticoagulants are administered at levels that effectively modulate hemostasis and reduce coagulation tendency [7,9].

To date, homologous VKOR enzymes have been detected in hundreds of prokaryotic and eukaryotic species through whole genome sequencing efforts. A number of representative VKOR enzymes from bacteria, plants and animals have been cloned and studied [7,9,10,11,12,13,14,15,16]. Surprisingly, bacterial and plant VKOR homologs studied so far have been shown to not possess K>O de-epoxidase (VKOR) activity and do not use K>O as a substrate. Alternatively, these enzymes possess only quinone reductase activity which reduces ubiquinone or K substrates to the respective hydroquinone forms. Thus, an updated analysis of VKOR oxidoreductase family phylogenetics and associated review of functional and structural similarities and differences is warranted.

**Figure 1:**
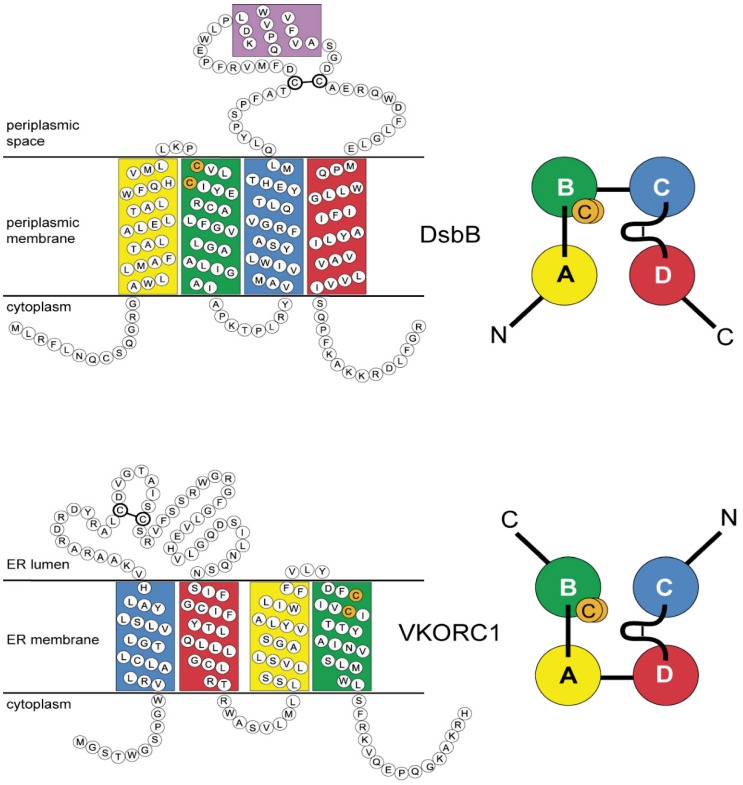
Relationship of structural elements for *E. coli* DsbB and human VKORC1. Single-letter amino acids (circles); transmembrane helices (four colored rectangles; coloring scheme according to [17]); two active site cysteines (orange circles); DsbB (top left) short helix (purple rectangle); two redox-active cysteines in DsbB large periplasmic loop (thick connected circles); Human VKORC1 (bottom left), transmembrane helix colors for homologous positions relative to DsbB. Transmembrane helical bundle organization and primary sequence threading (N, N-terminus; C, C-terminus) for DsbB (top right, view from periplasm) and VKORC1 (bottom right, view from ER lumen) are shown based on X-ray crystallographic structures. Letters A–D on the right side indicate helix designations used throughout the text. N- to C-terminus direction threading of the primary sequence through DsbB is “ABCD”, but “CDAB” for VKORC1; thus, both threadings are related to each other by circular permutation (CP). Structural elements for DsbB from PDB entry 2HI7 [29], for VKORC1 from PDB entry 3KP9 [14].

Structural and functional similarities among prokaryotic periplasmic and eukaryotic ER-resident oxidoreductase families involved in oxidative protein folding have been previously suggested, although lack of significant sequence homology suggests these families arose independently during evolution [17]. Specifically, these include Endopasmic Reticulum Oxidoreductin 1 (ERO1, PFAM PF04137, common to all eukaryotes), Augmenter of Liver Regeneration (Erv1/Alr, PFAM PF04777, common to all eukaryotes, some proteobacteria, cyanobacteria and all known cytoplasmic DNA virus taxa), and Disulfide Bond-forming protein B (DsbB, PFAM PF02600, common only to prokaryotes) families that share similar pairs of redox-active disulfide motifs [18]. Recently, studies confirmed a direct role for VKORC1 in oxidative protein folding in addition to its classically reported role in the vitamin K cycle which enables post-translational γ-glutamyl carboxylation of vitamin K-dependent proteins including a number of blood clotting factors [19,20]. VKORC1 is a representative of the vitamin K epoxide reductase family (VKOR, PFAM PF07884), named for the enzymatic function first identified in humans and rodents, namely, vitamin K 2,3-epoxide reductase (VKOR) activity, which is potently inhibited by warfarin and other 4-hydroxycoumarin derivatives [10,11]. Since the 1950s, warfarin and homologous 4-hydroxycoumarins have been the most widely prescribed oral anticoagulants to treat thromboembolitic diseases in humans and, at much higher, lethal dosages as rodenticides [21]. VKOR family sequences also possess two redox-active cysteine pairs and have been detected in taxa from the entire Tree of Life except for yeasts and fungi [22,23]. In prokaryotes, either VKOR or DsbB homologs are required for oxidative protein folding (Figure 1) [24,25]. Both DsbB and VKOR enzymes receive reducing equivalents via thioredoxin-like (Trx-like) oxidoreductases from pairs of redox-active cysteines during oxidative protein folding, ultimately passing the reducing equivalents to diffusible quinone cofactors. DsbB cofactors include ubiquinones (UQ) principally for aerobically respiring taxa and menaquinones (MK, K_2_ vitamins) for anaerobic respirants [26]. With the exception of only a very few obligate intracellular prokaryotic pathogens, all prokaryotic VKOR homologs apparently use UQ or MK, while plant VKORs use phylloquinone (PK, vitamin K_1_) as the sole electron accepting cofactor. All other eukaryote genomes from single-celled protists through higher invertebrates and vertebrates, but not those of fungi, encode VKOR homologs that can use MK or PK as cofactors, while jawed vertebrates (gnathostomes) can additionally use the more highly oxidized 2,3-epoxides of K vitamins which are naturally produced only in taxa possessing genomically encoded-glutamyl carboxlase homologs. While menaquinone 2,3-epoxides have been found in vertebrates, they are not known to exist in any prokaryotes [27,28].

Five studies have previously explored VKOR family phylogeny. Rost *et al*. (2004) reported cloning paralogous pairs of VKOR genes in human, rat, mouse and the fish Takifugu ruprides and identified VKOR homologs by gene annotations in the mosquito Anopheles gambiae, but not in the fruit fly Drosophila melangaster, suggesting that evolution of VKOR family proteins was later than the evolution of carboxylase enzymes for which *Drosophila* is known to possess a homologous gene [10]. They also noted the existence of two human VKORC1 retropseudogenes that arose independently. Goodstadt & Ponting (2004) presented a multiple sequence alignment analysis identifying VKOR homologs in 37 species of archaea, eubacteria, plants, invertebrates and vertebrates [22]. They further identified four conserved cysteines and a conserved serine/threonine as residues likely required for enzymatic function. Additionally, they noted that many plant and prokaryote VKOR homologs are multidomain proteins that often include a C-terminal thioredoxin-like domain in addition to the core VKOR domain. Using prediction algorithms, they also identified four putative transmembrane helices in the VKOR core domain. Indeed, a four-helical bundle organization of the VKOR domain was recently confirmed for a prokaryotic VKOR homolog by X-ray crystallographic analysis [14,30]. A study by Robertson (2004) confirmed that, while vertebrate genomes encode two VKOR paralogs, only single VKOR homologs are present in invertebrates, basal deuterostomes, protists and plants with no VKOR homologs detected in fungal taxa [23]. He also identified additional VKOR family retropseudogenes—three similar to human VKORC1L1, five similar to VKORC1L1 in mouse and three with VKORC1L1 similarity in rat. In contrast to the report by Rost *et al.*, Robertson was able to detect a VKOR homolog sequence in *Drosophila melanogaster* [10]. Robertson calculated an inferred phylogenetic tree with low bootstrap support using trypanosome protists as an outgroup, representing the most highly divergent homologs from higher animals. His results suggest that a gene duplication occurred resulting in VKORC1 and VKORC1L1 clades after vertebrates diverged from non-chordates. Schwarz *et al.* (2009) used profile hidden Markov models derived from multiple sequence alignments and correspondence analysis to distinguish between evolutionary and functional site-specific signals for VKORC1/VKORC1L1 paralog pairs of twelve vertebrate species [31]. Their analytical methods were able to distinguish functionally conserved and variable sites, detect clade-specific sequence clustering and revealed specific sites associated with clade splittings. Importantly, they identified the timing of the duplication event leading to VKORC1 and VKORC1L1 paralogs as occurring at the base of the vertebrate split from other metazoans. Finally, a recent study by Wan *et al.* (2014) that identified and characterized a VKOR family homolog in the tomato plant, presented evidence suggesting that all plant VKOR homologs possess an N-terminal signal peptide responsible for transit of the protein to inner thylakoid membranes and a C-terminal thioredoxin-like domain with four conserved cysteines [16]. They constructed a phylogenetic tree with 41 species that include plants, algae, photosynthetic bacteria and vertebrate animals. Vertebrates, monocot and dicot plants were segregated into three distinct clades, while conifers, mosses, cyanobacteria and green algae formed a weak clade with low branch support. Highest branch supports were for monocots and vertebrates.

In the present study, we reconstruct a comprehensive phylogeny for the VKOR family that includes 327 sequences from major representative phyla of the Kingdoms of Life and use the results to define major clades, explore clade-specific sequence differences that correlate with structural and functional differences of the clades, and propose an evolutionary scenario for the VKOR family that suggests an ancient common ancestor shared with the DsbB family.

## 2. Experimental Section

### 2.1. Protein Sequences and Multiple Sequence Alignments

Protein sequences were obtained from the NCBI Conserved Domain Database (http://www.ncbi.nlm.nih.gov/cdd/) and from the PFAM database (http://pfam.xfam.org) for VKOR (CDD: cl01729, VKOR Superfamily, 3748 raw sequences; PFAM: PF07884, VKOR family, 864 raw sequences) and DsbB (CDD: cl00649, DsbB Superfamily, 6673 raw sequences; PFAM: PF02600, DsbB family, 2959 raw sequences) families [32,33]. For each family, sequences were initially aligned using the MAFFT fast algorithm (FFT-NS-1 option) implemented in JalView2.8 by masking non-core domain sequence segments (core domains defined as DsbB (*E. coli*): Trp15-Ile162 and VKOR (*Synechococcus* sp.): Ile22-Val148 including first residue of first transmembrane helix to last residue of fourth transmembrane helix based on X-ray crystallographic data from PDB entries 2HI7 and 3KP9, respectively) [34,35]. Identical sequences from redundant species, partial or unrelated sequences not fully encompassing the core domain lengths, and sequences lacking known conserved residues were manually culled. Further reduction of sequences in over-represented phyla and classes resulted in final data sets of 327 non-redundant VKOR and 514 non-redundant DsbB sequences for phylogenetic analysis. The DsbB family multiple sequence alignment (MSA) was not subjected to further phylogenetic analysis. For the full-length (multidomain) MSA for the VKOR family, N- and C-terminal non-core domains were aligned independently of the core domain. Core domain alignment was iteratively refined based on WSP and consistency scores using MAFFT (G-INS-i option) and manually adjusted to align any obvious positionally conserved residues while minimizing the number of gapped segments. Additional sequence-truncated versions of each MSA included either only the core domains (defined above) or a 44 residue subsequence comprising the large loop joining the 1st and 2nd transmembrane helices of the VKOR core domain (“VKORloop”—delimited by VKORC1(*H. sapiens*): Asp36-Ser79). All multiple sequence alignment and tree files are included with the online Supplementary Materials and Research Data.

### 2.2. Phylogenetic Analyses

Maximum Likelihood (ML) analyses were conducted using the TOPALi2 ver2.5 (http://www.topali.org/index.shtml) and IQ-TREE ver1.2.3 (http://www.cibiv.at/software/iqtree/) server-based phylogenetic packages [36,37]. Phylogenetic model testing and selection included 40 distinct models by TOPALi2 and 150 models by IQ-TREE. Model evaluation was based on standard Akaike infomation criterion (AIC), corrected AIC (cAIC) and Bayesian information criterion (BIC) scores, where best models correlate with minimized scores. Preliminary ML phylogenetic tree reconstructions were estimated using either PhyML3.0 [38] or RaxML8.0.0 [39] with branch supports for 1000 bootstrap replicates calculated by the rapid UFBoot method [40]. The final VKORloop consensus tree was constructed from 1000 bootstrap trees using the IQ-TREE1.0 tree search algorithm [37]. Branch lengths were optimized by maximum likelihood on original alignment and bootstrap branch support calculated. To simplify visual presentation, the consensus tree was further pruned to 310 sequences using IQ-TREE1.0 and graphically rendered using the Interactive Tree of Life (iTOL) server (http://itol.embl.de) [41].

### 2.3. Assessment of Residue-Specific Evolutionary Conservation and Structural Correlates

For determination of residue-specific sequence conservation from VKOR and DsbB family MSAs, we used the ConSurf server (http://consurf.tau.ac.il) [42,43]. Briefly, ConSurf computes position-specific evolutionary rate inferences as continuous-variable conservation scores using the empirical Bayesian algorithm. The server creates an output text file where the continuous conservation scores are binned into nine grades for visualization, from the most variable positions (grade 1, colored turquoise), through intermediately conserved positions (grade 5, colored white), to the most conserved positions (grade 9, colored maroon). We mapped these conservation grades onto MSAs using JalView2.8 for analysis and graphical rendering. We further explored structural features corresponding to the conservation data revealed by ConSurf analysis by using the TMPad server (http://bio-cluster.iis.sinica.edu.tw/TMPad/) to visualize and determine residues involved in direct interhelical contacts from the representative X-ray crystallographic structures (PDB entries 2HI7 and 3KP9) [44].

### 2.4. Homology Modeling of Human VKORC1 and VKORC1L1 Paralogs Using a Cyclic Permutation of E. coli DsbB as the Target Structure

Homology models of human VKORC1 and VKORC1L1 were created using template sequences from the NCBI Protein database (Accession: Q9BQB6.1 GI: 62511226 and Accession: Q8N0U8.2 GI: 62511214, respectively) and *E. coli* DsbB (PDB entry 2K74, model 1 [45]) as template structure. The nearly identical lengths of DsbB (176 aa), VKORC1 (163 aa) and VKORC1L1 (176 aa) suggested to us that indels would not be a major concern in modeling. Briefly, we swapped the first and second halves of the VKORC1 and VKORC1L1 primary sequences in order to create an optimized alignment to the DsbB sequence (see Figures S1–S4 and detailed methods in the online Supplementary Materials and Research Data) and generated initial models using MODELLER 9v7 [46]. In order to restore the native primary sequences and threading, the best quality initial models were edited to join the N- and C-termini and create new termini at the loop between the second and third transmembrane helices (TMHs) using Coot 0.6-pre-1 [47]. The 50 residue loops between the first and second TMHs of the edited models were replaced with same-sequence loops *de novo* modeled using the I-TASSER server [48]. Addition of hydrogen atoms and quality assessment of final models was performed using the MolProbity server [49]. Final models were submitted to the Protein Model DataBase (VKORC1: PMDB entry PM0075969, VKORC1L1: PM007970; both models deposited 21 September 2009, public release 1 September 2010).

## 3. Results

### 3.1. Multiple Sequence Alignments Reveal Greater Inhomogeneity in Indels for the VKOR Family Core Domain Relative to the DsbB Family

Initial inspection of high quality multiple sequence alignments for the core domains of the VKOR family (327 sequences, truncated to residues corresponding to human VKORC1: Leu13-Phe150 in order to remove highly variable N- and C-terminal sequences) and DsbB family (514 sequences, truncated to residues corresponding to *E. coli* DsbB: Gly13-Pro165 in order to remove highly variable N- and C-terminal sequences) revealed a larger heterogeneity in indel sizes and locations for the VKOR core domain (10 major indels for >1 sequences, ranging 2–27 residues in length) relative to the DsbB core domain (5 major indels for >1 sequences, ranging 2–11 residues in length) (see online Supplementary Materials Figures S5 and S6 and Research Data for FASTA-formatted MSA and graphical summary files; only non-terminal indels were counted in order to avoid possible incomplete sequencing reads in the original data; minor indels comprising single residues were not included.) Thus, the greater overall sequence diversity for the VKOR family core domain suggests greater overall evolutionary diversity, relative to the DsbB core domain. This is consistent with the evolutionary limitation of DsbB family taxa to prokaryotes *versus* the expansion of the VKOR family taxa into all major Kingdoms of Life.

### 3.2. VKOR Family Phylogeny is Organized into Five Principal Clades

**Figure 2:**
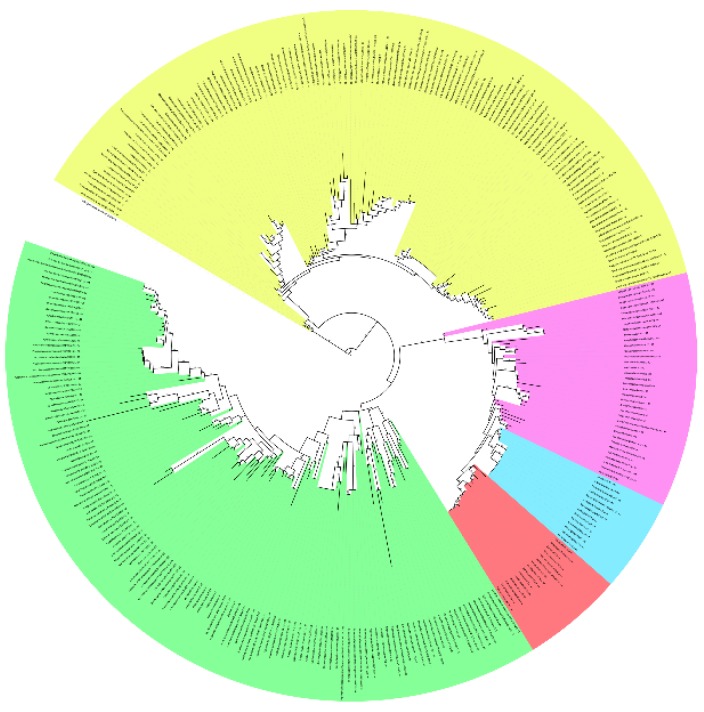
Maximum likelihood-estimated phylogeny for VKOR family based on VKORloop sequences for 327 non-redundant sequences (pruned to 310 sequences). Five major clades are shown in color (yellow, non-photosynthetic prokaryotes; green, photosynthetic prokaryotes and plants; magenta, primitive single-celled animals and invertebrates; blue, vertebrate VKORC1L1 homologs; red, vertebrate VKORC1 homologs. Tree is rooted using the archaeon *Caldivirga maquilingensis* as outgroup (single uncolored branch between yellow and green clades). Numbers at branches (see online Supplementary Materials and Research Data; visible when enlarged in PDF reader) indicate bootstrap supports (%). Accurate inclusion of all sequences in the VKORC1 and VKORC1L1 clades was manually verified despite incorrect annotations in some sequence database entries that led to false name assignments (e.g., where C1 and L1, representing VKORC1 and VKORC1L1, respectively, are indicated after the genus/species names). A PDF file of this figure is included with online supplementary data for enlarged viewing.

Testing of standard phylogenetic amino acid substitution models using both TOPALi2 and IQ-TREE for the VKOR family MSA using 327 non-redundant full-length sequences resulted in the Whelan & Goldman rate heterogeneity model with invariant sites as the best model (see online Supplementary Materials and Research Data for sequences, MSAs and substitution model statistics). Subsequent phylogeny reconstruction using PhyML or RaxML revealed unrooted trees with five large cladistic groupings, although two clades included mixed prokaryotic, invertebrate and vertebrate VKOR homologs and animal sequences were found among the predominantly bacterial and plant clades. Similar results were obtained, with increased mingling of prokaryotic sequences among the predominantly invertebrate and vertebrate clades, when we truncated the MSA to include only the VKOR core domain (defined in Section 2.1). Previous reports have suggested that TMHs considerably contribute to phylogenetic noise, due to high frequency of occurrance and degeneracy among nonpolar residues in the hydrophobic membrane core and a relatively low selection pressure on residues that face into the lipid bilayer [50,51]. Also, a phylogenetic study of DsbB family homologs reported difficulties in automated sequence alignments due to amino acid compositional bias in the transmembrane region [52]. Accordingly, to enhance phylogenetic signal-to-noise levels for the VKOR family, we chose to further truncate the MSA to the large loop segment between the first two TMHs for two principal reasons: (1) the loop includes three out of five conserved residues known to be essential to VKOR enzymatic activity and other residues in this segment may also contribute to substrate specificity and/or catalysis; and (2) the loop is known to bind to and accept reducing equivalents from species-specific partner oxidoreductases essential for VKOR enzymatic function *in vivo* [53]. Thus, the loop-specific sequences (50 residues) encode a large amount of evolutionary information pertinent to enzyme function. Indeed, phylogeny reconstruction using the VKORloop sequences (corresponding to human VKORC1: Asp36-Ser79) and the best model (WAG + I + G) yielded a consensus tree with well-defined clades consistent with proposed versions of the Tree of Life based on, for example, ribosomal protein or tRNA synthase phylogeny (Figure 2) [54,55,56,57].

**Figure 3:**
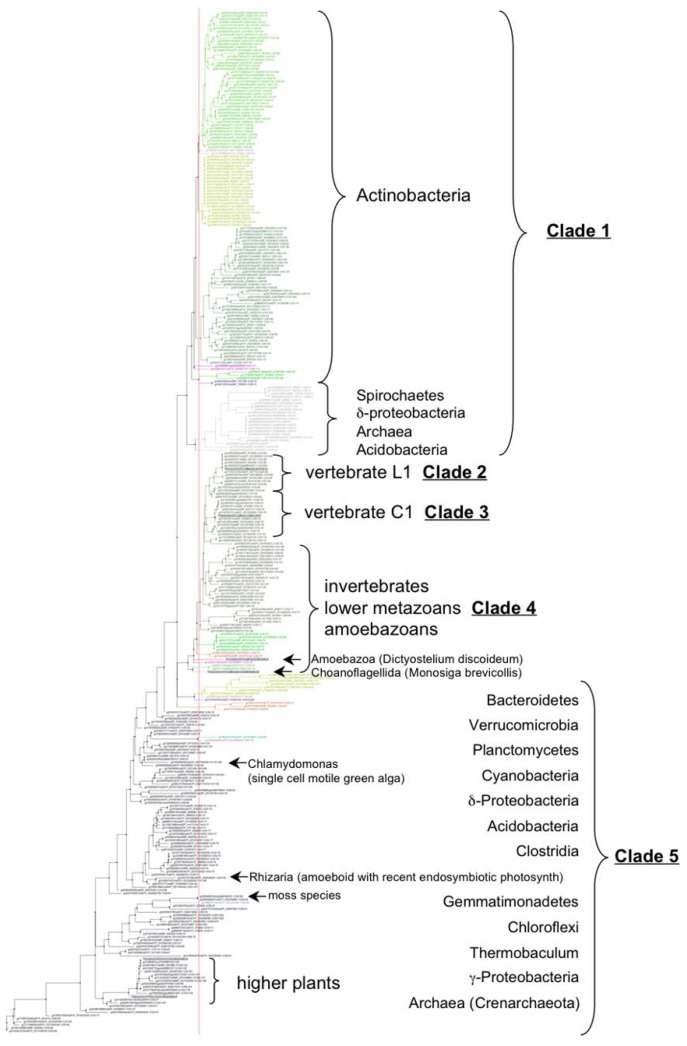
Cladogram (unrooted tree) of VKORloop sequences identifying groupings into highest common Linnaean taxonomic ranks. (See online supplemental data for a complete listing of protein identifiers in the FASTA-formatted MSA file; also, Figure S7 of the online Supplementary Materials and Research Data shows the full-length aligned sequences, including non-VKOR domains, corresponding to the cladogram in this figure).

Figure 3 summarizes the major taxonomic classes and phyla comprising the five clades for the 327 non-redundant sequences. Clade 1 (Figure 2, colored yellow) is polyphyletic and includes non-photosynthetic archea and eubacteria. Actinobacteria, one of the three major Gram-positive phyla, comprise the major grouping along with acidobacteria, crenarchaeota (including species of the *Caldivirga*, *Pyrobaculum* and *Thermoproteus* genera grouped together in a discrete subclade), proteobacteria and spirochete representatives. All actinobacteria possess a predicted fifth transmembrane helix (TMH), but no C-terminal Trx-like domain, whereas proteobacteria and spirochetes possess both a fifth TMH and C-terminal Trx-like domain. Clades 2 and 3 (Figure 2, blue and red, respectively) are both individually and together monophyletic and include members of the vertebrate VKORC1 and VKORC1L1 paralogs, respectively, as single VKOR domains. Clade 4 (Figure 2, magenta) is paraphyletic and comprises single-domain VKOR homologs encoded in genomes of non-vertebrate animals including amoebazoans, choanoflagellates and lower metazoans through invertebrates. Clade 5 (Figure 2, green) is polyphyletic and comprises archaea, eubacteria and eukaryotes capable of various forms of photosynthesis from green sulfur-metabolizing hyperthermophilic prokaryotes through algae and higher plants. Bacteriodetes, Chloroflexus and Roseiflexus taxa all have additional N-terminal domains. Trx-like CXXC signature motifs are found in only the Chloroflexus and Roseiflexus N-terminal domains and these taxa lack C-terminal Trx-like domains. All other clade 5 taxa possess C-terminal Trx-like domains except for Archaea and Gemmatimonadetes. Interestingly, Clade 5 includes intermingled archaeal and eukaryotic taxa, suggesting horizontal gene transfer (HGT): a cluster of three archaeal *Pyrobaculum* species, all different from those in Clade 1, appear along with a single Verrucomicrobia representative (genus *Chthoniobacter*), the only non-photosynth grouping in Clade 5 (there are, however, other single Verrucomicrobia sequences intermingled among various Clade 5 branches, further suggesting HGT); a single Rhizaria eukaryote, *Paulinella chromatophora*, is found on a Clade 5 branch between branches bearing *Synechococcus* species. *P. chromatophora* is a freshwater amoeboid widely noted for its very recently acquired cyanobacterial symbiont previously believed to stem from an ancestor of either *Synechococcus* or *Prochlorococcus* genera [58]. Compared to free living *Synechococcus* species, the *P. chromatophora* plastid has retained ~26% of the original genome (*i.e.*, retained 867 protein-coding genes), although the VKOR family homolog (NCBI Protein: YP_002048937.1) was previously transferred, along with most of the plastid genes, to the *P. chromatophora* nuclear genome [59]. Thus, our reconstruction of the VKORloop phylogeny strongly suggests that the *P. chromatophora* chloroplast evolved from an acquired *Synechococcus* endosymbiont, further resolving the uncertainty of a phylogenetic reconstruction study by Marin *et al.*, based on analysis of concatenated rDNA operon or rRNA sequences, to determine if the ancestral endosymbiont was an ancestor of *Synechococcus* or *Prochlorococcus* [60].

### 3.3. Similarities and Differences among Sequence Position-Specific Substitutions Reveal VKOR Family Clade-Specific Structural and Functional Residues

**Figure 4:**
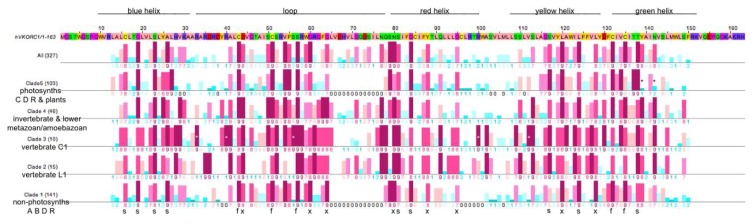
Comparison of Consurf conservational scoring for combined VKOR family sequences and the five individual VKOR clades. Human VKORC1 primary sequence is with relative postions of transmembrane helices (indicated using color scheme from [17]) and large loop as line segments (top); calculated Consurf conservation scores on lines below the sequence (six lines, top to bottom): All 327 VKOR sequences; Clade 5, 103 photosynthetic prokaryotes of the super-taxa C, D and R (prokaryotic super-taxa: A = Actinobacteria, B = Bacilli an related, C = Clostridia and related, D = double membrane Gram-negative bacteria, R = Archaea [56]); Clade 4, 46 invertebrates, lower metazoans, euglenozoans and mycetozoans; Clade 3, 10 vertebrate VKORC1 orthologs; Clade 2, 15 vertebrate VKORC1L1 orthologs; Clade 1, 141 non-photosynthetic prokaryotes of the super-taxa A, B, D and R. Darker red bars indicate greater positional conservation, darker blue bars indicate greater positional non-conservation (conservation scores below bars; lowest score 1 to highest score 9). Systematically absent from the prokaryotic sequences (clades 1 and 5) indicated with black zeros in place of Consurf scores. Fully conserved vertebrate VKORC1 residues (white asterisks superimposed upon dark red bars); Clade 5-specific absences of conserved Tyr139 and Asn142 (black asterisks above dark blue bars, human VKORC1 numbering). Letters and symbols (bottom) for positions highly conserved across all five major clades: s = structural residue contributing to helix-helix packing interactions; f = residues full conserved across all species and presumed to be catalytically active (Cys43, Cys51, Ser57, Cys132, Cys135); x = residues that are highly conserved, but presently lacking assignment as to structural or functional importance.

In order to explore VKOR family clade-dependent and clade-independent sequence conservation, and to assess if conserved sites might have functional or structural correlates, we performed site-specific conservational analyses on the complete MSA as well as on the aligned sequences for each of the five identified VKOR family clades. Figure 4 graphically summarizes the conservation analysis results.

We identified 22 residue positions (Figure 4, bold black letters **s**, **f**, **x** below the Clade 1 line) that are highly conserved across all VKOR clades. We have assigned structural and functional roles to these positions as follows:

(1) The five fully conserved VKOR signature residue positions (Figure 4, marked **f**) are functional, playing direct roles in vitamin K 2,3-epoxide to vitamin K quinone and vitamin K quinone to vitamin K quinol reduction (Cys43, Cys51, Ser57, Cys132, Cys135 according to human VKORC1 numbering);
(2) Nine highly conserved positions (Figure 4, marked **s**) represent residues that form putative helix-helix structural contacts identified by visual inspection of the X-ray crystallographic structure for the prokaryotic VKOR homolog (PDB entry 3KP9, residue numbering corresponds to human VKORC1 sequence in Figure 4). On TMH1, Cys16, Gly19, Ser23 and Ala26 pack against TMH2, TMH2 and TMH4, TMH2, TMH2 and TMH3, respectively. On TMH2, Ser81 and Gly84 both pack against TMH1. On TMH3, Ser117 packs against TMH4, while Leu124 packs against only residues on the same helix (TMH3). On TMH4, Thr138 packs against TMH1;
(3) Eight highly conserved positions (Figure 4, marked **x**) may be putative functional residues either essential for quinone substrate reduction or involved in substrate binding and specificity (Asp44, Gly80, Gly84, Asn80, Tyr88, Gly95, Leu120, Leu128).

Notable differences in position-specific conservation between VKOR family clades include:

(1) Compared to non-animal VKOR homologs, there are conserved sequence insertions among animal VKOR homologs (clades 2–4; Figure 4, absence of animal-specific sequences indicated as conservation scores “0” for clades 1 and 5). A few of these insertions are individual or adjacent pairs of residues located in two groupings—one at the margin including the C-terminal portion of TMH1 and the large loop joining TMH1 and TMH2, and the other in the C-terminal margin of TMH2 and the variable-sized loop connecting TMH2 and TMH3. The largest insertion is a linear sequence of 13–14 residues that comprise and extension at the C-terminal end of the large loop which joins TMH1 and TMH2. Thus, these animal insertions positively correlate with the representative homologs possessing VKOR (*i.e.*, phyloquinone- and menaquinone-2,3-epoxidase) catalytic activity in addition to general quinone (*i.e.*, menaquinone, phyloquinone or ubiquinone) reductase activity common to all VKOR family members. To-date, with only the exception of demonstrated *in vitro* VKOR activity for two obligate intracellular bacteria that are pathogenic in eukaryotes (*M. tuberculosis*, *Corynebacterium jeikeium*) [13], all representative non-animal VKOR enzymes for which quinone substrate usage has been biochemically characterized were found to lack VKOR activity (eubacteria: *Synechococcus* species [14]; *Synechocystis* sp. PCC6803 [61]; *Roseiflexus* sp. RS-1, *Salinispora tropica* CNB-440 [13]; plants: *Arabidopsis thaliana* [15], *Solanum lycopersicum* [16]).

(2) A site-directed mutagenesis study by Matagrin *et al.* (2013) of rat VKORC1 revealed Tyr139 (homologous residue for human VKORC1 is also Tyr139) to be critical to VKOR catalysis in that removal of a 3-hydroxy group from vitamin K, once the epoxide oxirane ring has been opened, is apparently hindered by substitution of the tyrosine by cysteine, phenylalanine or serine as in naturally occurring warfarin resistant VKORC1 variants in rats [62]. In agreement with their results, a previous study also reported the production of a proposed VKOR catalytic intermediate product, 3-hydroxyvitamin K, by liver microsomes from a homozygous warfarin-resistant HW Welsh rat strain later genotyped as VKORC1:Tyr139Ser [63,64]. We find that tyrosine is completely conserved at the primary sequence position homologous to human and rat Tyr139 among all metazoan VKOR family homologs belonging to Clades 2, 3 and 4. Alternative substitutions at this positions include chiefly tryptophan (infrequently, methionine or threonine) among Clade 1 homologs, while Clade 5 substitutions include chiefly residues with aliphatic side-chains (*i.e.*, Ala, Leu, Ile). Interestingly, the only Clade 1 or 5 homologs with Tyr at this position include prokaryotes of the genera *Leptospira* (spirochetes) and *Streptomyces* (filamentous soil bacteria), *Leishmania* (a genus of trypanosomatid protozoa) and *Trypanosoma* (a genus of kinetoplastid protozoa), all obligate intracellular pathogens in vertebrates, suggesting that these VKOR family homologs might catalyze VKOR activity. At least nine *Streptomyces* species have been reported to be pathogenic in humans (55) [65]. Also, *Leptospira interrogans* is known to possess a gene for a glutamyl carboxylase homolog presumed to have been obtained through HGT, lending support to the possibility that the genomically encoded VKOR family homolog enables production of vitamin K-dependent proteins involved in pathogenesis (56) [66]. For identification purposes, we name this the *LSLT* group of pathogenic organisms.

(3) We identified a covariant site for the *LSLT* pathogenic group at the residue homologous to human VKORC1: Ile136 immediately following the conserved active site CXXC motif. Species in the *LSLT* group possess a cysteine at this position homologous to the human sequence numbering VKORC1: Ile136Cys and include *T. brucei*, *L.* major strain Friedlin, *L. infantum*, and *Streptomyces* species (*coelicolor A3(2), griseus* sub.sp. *griseus*, *sviceus*, sp. SPB74, and two distinctly non-identical VKOR family homologs for sp. Mg1) from our MSA data set. We could not identify any additional position-specific markers in the VKOR core domain for this group.

A notable, distinguishing feature of all vertebrate VKORC1 and VKORC1L1 sequences (clades 2 and 3) is the systematic absence of segments of the large loop region from the prokaryotic sequences (Figure 4; clades 1 and 5, indicated with black zeros among the Consurf scores). Thus, both prokaryotic clades are missing the equivalent of human VKORC1 sequence residues Leu65 through Asn77 that comprise the distal C-terminal residues of the large loops in clade 2, 3 and 4 sequences. Differential clade-specific absences of additional sequence segments occur in the N-terminal portion of the loop as well as in regions comprising portions of the red and yellow helices and adjoining small cytoplasmic loop for the prokaryotes (Figure 4; clades 1 and 5, indicated by black zeros). Vertebrate VKORC1 (clade 3-specific) full conservation is indicated in Figure 4 by white asterisks superimposed upon the highest conservation score bars (dark red) at Arg33, Arg40, Ser56, Arg100 and Val112 (human VKORC1 numbering). Interestingly, lack of clade 5-specific conservation at Tyr139 (human VKORC1 numbering), as well as at Asn142 (one helical turn away from Tyr139) indicates non-conservation at these positions, in striking contrast to conservation at these positions in the other four clades.

### 3.4. Evidence for an Evolutionary Relationship between VKOR and DsbB Families

**Figure 5:**
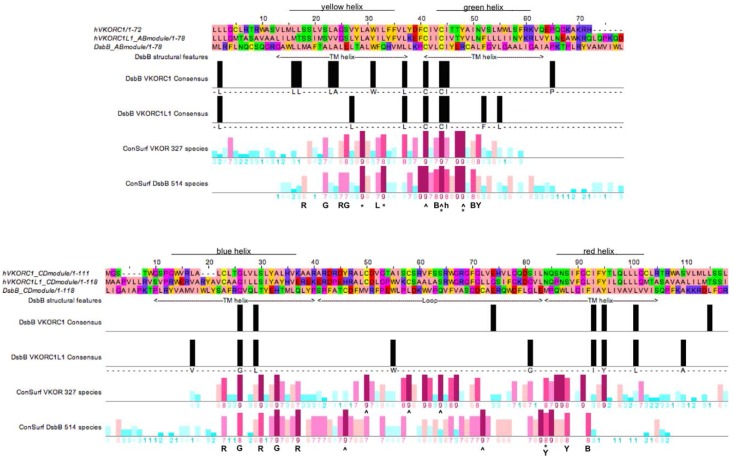
Alignment of helix pair-loop module sequences of VKORC1, VKORC1L1 and DsbB showing pairwise identity, Consurf conservational scoring and known DsbB secondary structure elements and interhelical packing residues (Note 1 [67]). ClustalW2 alignments for AB (top) and CD (bottom) modules. Positions of transmembrane helices for DsbB are indicated below alignment, helix color indicated above alignment. Vertical black bars, sequence identity between DsbB and VKOR proteins; bottom two lines, Consurf conservation scores for VKOR multiple sequence alignment (second from bottom line) and DsbB multiple sequence alignment (bottom line). Vertical colored bars, positional conservation as described in Figure 4—Letters and symbols below conservation score lines: R = interhelical contact to red helix, G = interhelical contact to green helix, B = interhelical contact to blue helix, Y = interhelical contact to yellow helix, * = residue making contact with quinone cofactor, L = residue mediating hydrophobic packing against a C-terminal segment of the large loop, ^ = residues completely conserved in all species, h = residue making hydrophobic contact with the small amphipathic helix of the large loop.

In order to investigate our hypothesis that the VKOR and DsbB families may have evolved from a common ancestor, we explored sequence similarity and conservation by creating aligned Consurf profiles for homologous structural elements from each family based on the availability of high-resolution X-ray crystallographic structures for the representative *Synechococcus* sp. VKOR and *E. coli* DsbB homologs. Specifically, we aligned two different conservation profiles representing helix pair-loop modules. The first (AB) module encompasses the primary sequences including the first two TMHs of VKOR and the last two TMHs for DsbB and including the intervening large loops (Figure 5, upper half). Accordingly, the second (CD) module encompasses the last two TMHs of VKOR and the first two TMHs of DsbB, together with the respective short connecting loops (Figure 5, lower half). As individual sequences among the VKOR and DsbB families have variable lengths due to species-specific indels, we truncated the family MSAs to include only sites of the primary sequences of the human VKORC1 and VKORC1L1 paralogs and *E. coli* DsbB homolog. To align the VKOR and DsbB family conservation profiles for each module, the representative human and prokaryotic index sequences were translated relative to each other until the invariant CXXC motifs were aligned for the AB module; the CD module was aligned by assessing pairwise identity between the VKORC1 and DsbB index sequences and between the VKORC1L1 and DsbB index sequences (Figure 5, black bars). Altogether, the best AB module alignment resulted in a maximum of five identity sites among the three index sequences; the best CD module alignment resulted in a maximum of four identity sites among the three index sequences (Figure 5, for each upper and lower figure halves, compare black bars on both pairwise identity lines). Inspection of the aligned positional conservation scores representing 327 VKOR homologs and 514 DsbB homologs reveals a very strong correlation in position specific conservation (compare patterns of dark red bars representing high Consurf scores).

In addition to the interfamily conservation of the active site CXXC motifs in the AB module for both families, we could assign conserved interhelical contact residues for the DsbB family: five helix-helix contacts (HHCs) for the A helices, three HHCs for the B helices (by inspection of the best high-resolution NMR structure; PDB entry 2K74). Of these DsbB family AB module HHCs, there is a perfect match (Figure 5, top half, AB module alignment position 26) corresponding to an HHC with a Consurf score of 8 at the corresponding human VKORC1:S117 position in the A helix (see Figure 4) representing an HHC also common to all VKOR family members. Similarly, at AB module position 33, the DsbB family conserved HHC matches a VKOR family conserved HHC with a Consurf score of 9 at the corresponding human VKORC1: Leu124 position in the A helix. For the B helices of both families, a conserved HHC at VKORC1: Thr138 with Consurf score 9 matches a similar highly conserved HHC in the DsbB family (Figure 5, upper half, AB module position 47).

For the CD module, the C helix of the DsbB family has five conserved HHCs, of which four VKOR family conserved HHCs including VKORC1: Cys16 (CD module position 23), VKORC1: Gly19 (CD module position 26), VKORC1: Ser23 (CD module position 30) and VKORC1: Ala26 (CD module postion 33) are perfect matches. The DsbB family D helix has three conserved HHCs, for which one VKOR family conserved HHC (VKORC1: Ser81, CD module position 88) is a perfect match.

Thus, independent of different threading of primary sequences through the helical bundle for each family (*i.e.*, N- to C-terminus threading comprises helices ordered ABCD for the DsbB family, but CDAB for the VKOR family), a highly conserved network of altogether 8 interhelical structural contacts (3 AB module and 5 CD module HHCs) is shared by both VKOR and DsbB family protein folds.

Similarly, when we surveyed the available high-resolution structural data for position-specific matches between VKOR and DsbB residues interacting with bound ubiquinone substrates, out of a total of 9 DsbB residues contacting a bound ubiquinone-2 and 18 VKOR residues contacting a bound ubiquinone-4, we found conserved matches at AB module positions 25, 29, 33, 44 for both VKOR and DsbB families. The greater number of ubiquinone contacts for *Synechococcus* sp. VKOR compared to those for *E. coli* DsbB is primarily due to the longer isoprenoid chain length of ubiquinone-4 *versus* that of ubiquinone-2. Of the four positions common to ubiquinone binding for both representative high-resolution structures, position 44 represents one of the conserved active site cysteines in the CXXC motif (homologous to human VKORC1: Cys135) located on helix B, while positions 25 (Consurf scores 6 and 7), 29 (Consurf scores both 6) and 33 (Consurf scores 8 and 9) represent conserved residues on the adjacent helix A. We interpret these findings to be highly supportive for conservation of key homologous substrate binding site residues among VKOR and DsbB families. Taken together, we believe these results constitute substantial evidence for structural feature conservation for both VKOR and DsbB families in support of our hypothesis that both families share a common ancestor.

### 3.5. Identification of Transmembrane-Helical Bundle Protein Families Related by Cyclical Permuted Primary Sequences Can Extend Homology Modeling into the “Twilight Zone” (<30% Sequence Homology between Target and Template)

**Figure 6:**
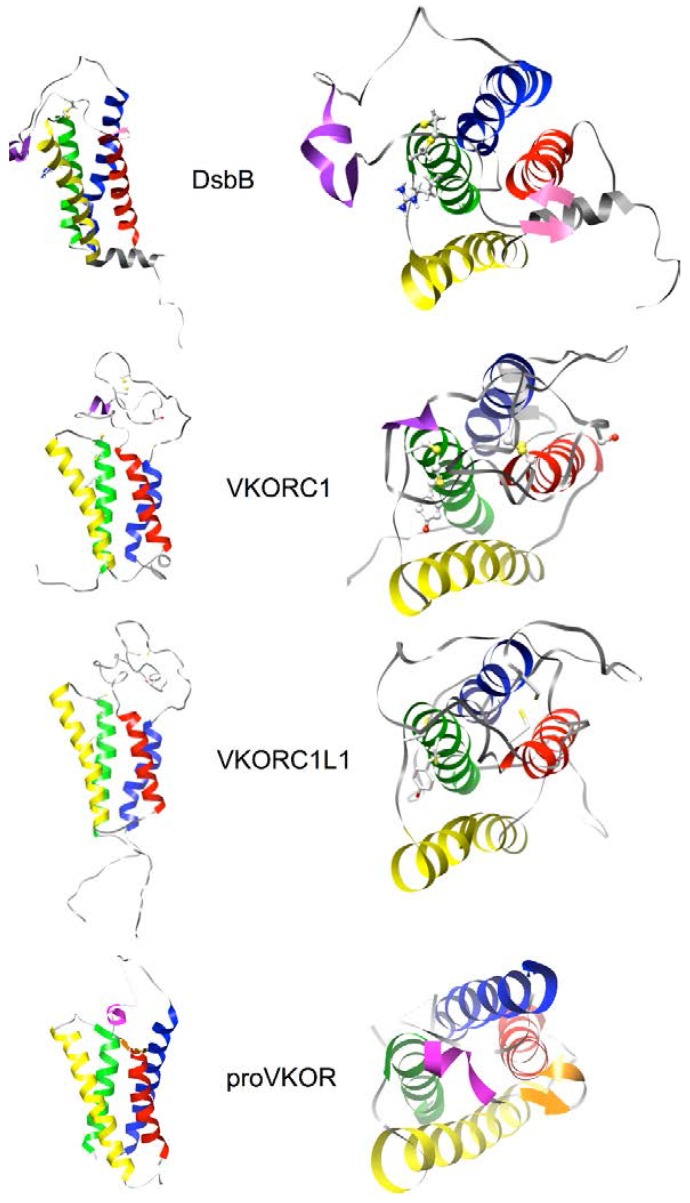
Backbone ribbon renderings of DsbB (PDB entry 2K74, model 1) [45], homology models of human VKORC1 and VKORC1L1 (Note 1 [67]), and Synechococcus sp. VKOR (proVKOR; PDB entry 3KP9) [14]. Views in the plane of the lipid bilayer (left) and normal (right) to the membrane plane from the ER lumenal (for VKORC1, VKORC1L1, proVKOR) or periplasmic (for DsbB) side. Transmembrane helices are colored (from N- to C-terminus) blue, red, yellow, green (designated as A, B, C and D, respectively, throughout the article text). Short loop helices for DsbB and VKORC1 (purple), for proVKOR (magenta); short β-sheet are regions (magenta or orange arrows); loop cysteines (ball and stick side-chains with yellow sulfur atoms).

Figure 6 shows the human VKORC1 and VKORC1L1 models we produced along with the NMR structure of the DsbB template and the X-ray crystallographic structure of the prokaryotic VKOR family homolog (proVKOR) by Li *et al.* (2010) [14]. Stereochemical validation performed on both models indicated overall quality similar to that of the X-ray crystallographic and NMR structures (see online Supplementary Material & Experimental Data). Overall organization of the transmembrane helical bundles is quite similar between the DsbB and proVKOR experimentally determined structures, although the large loops (at the top of each structure) exhibit major structural differences. For DsbB, the loop extends across the bundle and is thought to be anchored in the lipid membrane by a short amphipathic helix (shown in purple). In contrast, for proVKOR, the loop is positioned entirely above the bundle (note that several short segments of the loop were not resolved in the crystallographic structure and are shown as dashed grey lines in Figure 6) with a short helix serving as a cap centered directly over the bundle (shown in magenta). The VKORC1 and VKORC1L1 models have transmembrane helical bundles organized very similarly to those of proVKOR and DsbB. Noticeably, the large loops of the models do not appear structurally homologous to that of DsbB because they were modeled separately using *de novo* modeling methods in order to avoid bias favoring structural features of the DsbB template loop region. The *de novo* modeling methods we used (see Experimental Section 2.4) identify pieces of structures from the PDB to use as templates for building up a realistic protein structure based on threading short segments of the target sequence. The resulting loop models were computationally annealed as small globular domains and could be easily fit to the respective helical bundles using standard X-ray crystallographic model building software. Interestingly, the *de novo* modeled loops for VKORC1 and VKORC1L1 prominently exhibit short helices located at nearly the same position in the final models as the short capping helix of the experimentally determined proVKOR structure (Figure 6, purple helix shown in the loop for VKORC1, for VKORC1L1 the helix is visible, but not colored, at the equivalent position). Taken together, the similarity between the experimental proVKOR loop and our modeled loops for VKORC1 and VKORC1L1 suggested to us that the large VKOR loops, in general, are likely to behave more like well-folded, small globular protein domains than the extended loop observed for the DsbB structure. Another potentially realistic feature of our modeled loops is that, for both VKORC1 and VKORC1L1, the loop cysteines appear to be within disulfide bonding distance from each other and, additionally, located at the very top periphery of the protein where they would theoretically be accessible for binding to, and for reduction by, ER lumenal partner oxidoreductases.

## 4. Discussion

**Figure 7:**
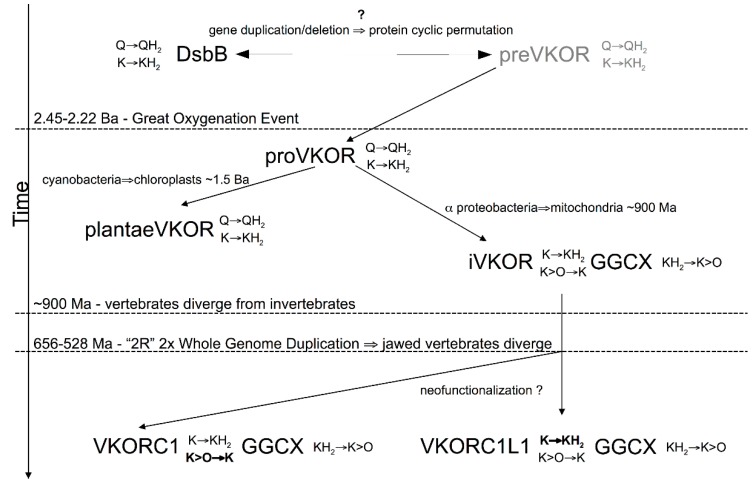
A comprehensive model for the evolution of the VKOR family and its hypothesized relationship to the DsbB family, suggested by the close relationship between the structure of the present reconstructed VKOR family phylogeny and that of the current consensus for the Tree of Life. Abbreviations and symbols (except if previously introduced): Ma, million years ago; Ba, billion years ago; Q, ubiquinone; QH_2_, ubiquinol; K>O, vitamin K 2,3-epoxide; K, vitamin K quinone; KH_2_, vitamin K hydroquinone; preVKOR, last universal common ancestral VKOR; proVKOR, prokaryotic VKOR; iVKOR, invertebrate VKOR; plantaeVKOR, plant VKOR; GGCX, γ-glutamyl carboxylase.

Reconstruction of VKOR family phylogeny by standard ML methods revealed five distinct clades that follow a rationale consistent with evolution based on standard Tree of Life models. Analysis of overall VKOR family-specific and individual clade-specific sequence variability further revealed conserved sequence motifs and individual residue positions that suggest a possible evolutionary relationship with the DsbB family. Accordingly, results from our analysis of interhelical packing residues for representative experimentally determined structures from both families lends support to this hypothesis. Furthermore, differences in sequence conservation between VKOR clades have provided useful information for deducing a plausible evolutionary chronology for the VKOR family (Figure 7).

Of potentially broader implication to the structural biology of membrane proteins is the first discovery of cyclic permutation of sequence threading through seemingly unrelated protein folds for helical transmembrane proteins. In the case of the VKOR and DsbB families, there has been enough preservation of common structural elements for both protein fold and substrate binding, as well as preservation of enzymatic function, substrate class and functional partner proteins, to reveal a plausible underlying evolutionary relationship. Using phylogenetic conservation data from large multiple sequence alignments for both families, despite only 12.0%–13.6% shared primary sequence identity between the human VKOR paralogs and *E. coli* DsbB, we have presented the first evidence for evolutionary relatedness between these families.

Overall, polytopic membrane protein structures solved by X-ray and electron crystallographic and NMR methods account for <1% of Protein Data Bank entries [68], yet represent up to an estimated 30% of genomic proteins and include upwards of 70% of known or potential therapeutic drug targets [69,70]. Identifying distant sequence homology targets for polytopic membrane protein templates with solved, high-resolution structures would prove beneficial to modeling efforts aimed at increasing structural coverage and would aid in defining functions for the rapidly growing number of new gene sequences from large-scale genomics efforts. Structural genomics aims to generate a minimum number of experimental structures to encompass all structural folds, including the subset of an estimated 550 (90% coverage) unique polytopic membrane protein folds [71]. Template-based models (TBMs) can be routinely constructed for target sequences sharing greater than 30% identity with an identified structural template [72] and used to guide hypothesis-driven experimentation [73] to study function [74] as well as to aid model building for crystallographic [75] and NMR data [76,77]. However, target sequences with less than 30% identity to any known template are considered to be in the “twilight zone” of template-based modeling [72,78], the case being exacerbated for membrane proteins where there are relatively few high-resolution structures [79].

*E. coli* DsbB and human VKORC1, for which experimental structures have been solved [14,29,30,45,80,81], share strikingly similar size, topology, two completely conserved functional cysteine pairs, lipidic quinone cofactors and oxidoreductase binding partners [19]. Moreover, primary sequence threading through the transmembrane α-helical secondary structural elements of both proteins is related by circular permutation (CP). Since 1979, there have been reports of CP relating the structural organization of naturally occurring soluble proteins (for a recent review see [82]) and underlying genetic principles leading to such permutations have been studied in the context of protein fold evolution [83]. We performed a literature-based survey of all published reports of CP in proteins and found examples for soluble protein domains, soluble domains of monotopic membrane proteins, sandwich transmembrane toxins, and a surprising number of engineered proteins including soluble domains as well as barrel transmembrane proteins occasionally genetically engineered to overcome crystallization problems for X-ray structural determinations. However, we could not identify any reports of CP for polytopic helical membrane proteins.

We advance the hypothesis and provide supporting evidence that both VKORC1 and DsbB families are evolutionarily divergent members of a single lipidic quinone:disulfide oxidoreductase superfamily (we suggest the nomenclature “LQOR superfamily”) structurally related by CP threading homology that likely arose from an ancient gene duplication/deletion event. This event apparently occurred before eukaryotes evolved. It is likely that both families arose from a common ancestor in prokaryotes from a whole gene (ABCD) duplication that resulted in a concatenated linear tandem repeat (ABCDABCD), followed by deletions of the outer flanking helix pairs (ABCDABCD) to yield the CP-related secondary structure threading (CDAB). Unfortunately, this scenario confronts us with essentially another version of the “chicken or the egg” causality dilemma—we have no way of currently deducing which permutation, and hence which family, predates the CP event. However, considering cumulative results from genetic and biochemical studies to date, we may deduce that, whereas the enzymatic function of DsbB family members has remained rather constant over long evolutionary timescales and has remained exclusively restricted to prokaryotes, multiple *de novo* enzymatic functions have arisen for members of the VKOR family since the divergence of eukaryotes and prokaryotes (Figure 7). Furthermore, it seems reasonable that neofunctionalization of VKOR family enzymes to catalyze de-epoxidation likely did not first arise before the onset of the Great Oxygenation Event.

## 5. Conclusions

In the present report, we have updated the phylogenetic characterization of VKOR family proteins to include broader representative taxa from the entire Tree of Life than was possible only a few years ago. We hope these results will serve as a basis to further explore fundamental relationships between available genetic data and structure/function correlates for the VKOR family.

Moreover, our results suggest a generalized approach for detecting further distant protein homologs related by cyclic permutation of a primary sequence. Our results also suggest that pairs of transmembrane helices (TMHs) together with the connecting loop may form key minimal modular building blocks that enable such evolutionary fold changes for helical intrinsic membrane proteins.

We hope our results will encourage more researchers to strive to better understand the molecular-level function of the modern VKOR enzymes from each of the major clades and what roles they play in development, growth and homeostasis. Advances on these fronts will further help to understand the evolution of these only recently discovered enzymes.

## Supplementary Files

Supplementary File 1
