# Supplementary material for: Phylogeny of the Vitamin K 2,3-Epoxide Reductase (VKOR) Family and Evolutionary Relationship to the Disulfide Bond Formation Protein B (DsbB) Family
Source: Nutrients. 2015 Jul 29;7(8):6224–49. doi: 10.3390/nu7085281 (PMC4555120; doi:10.3390/nu7085281)
Supplement: Supplementary File 1 [file nutrients-07-05281-s001.docx]

Supplementary Information

1. ClustalW (version 2.0.12) alignments

human VKORC1 to *E. coli* DsbB: 22 identical of 162 aligned residues = 13.6 % Identity


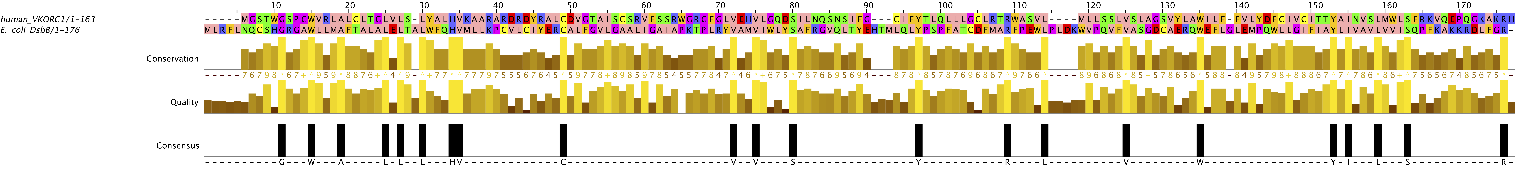


human VKORC1L1 to *E. coli* DsbB: 21 identical of 175 aligned residues = 12.0 % Identity


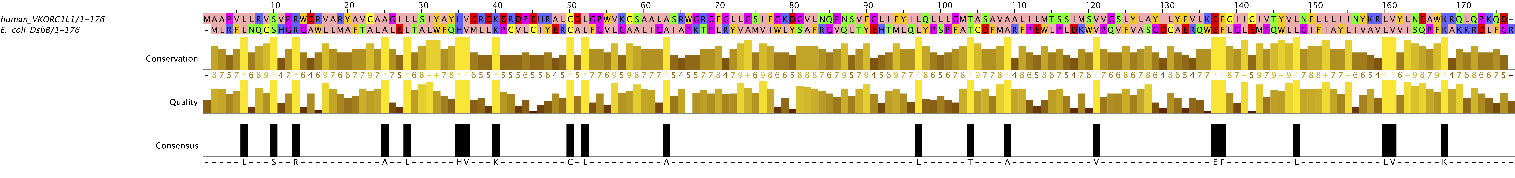


2. Sequence data

Multiple sequence alignments in FASTA format (.fa) and tree reconstructions in Newick (also called New Hampshire) format (.newick) files included in ZIP-formatted folder “MSAs & trees.zip”:

DsbB nr 514 full-sequences MSA.fa

VKOR nr 327 full-sequences MSA.fa

VKOR nr 327 full-sequences PhyML tree.newick

VKOR nr 327 domain22-148_SsVKOR MSA.fa

VKOR nr 327 domain22-148_SsVKOR PhyML tree.newick

VKOR nr 327 loop36-79_HsVKORC1 MSA.fa

VKOR nr 327 loop36-79_HsVKORC1 PhyML tree.newick

3. Amino acid substitution model results

VKOR nr 327 full-sequences MSA.fa: TOPALi2: WAG + I + G, Γ(α) = 2.141, ρINV = 0.019; quality assessment scores: *l* = −8844.32, AIC_1_ = 18994.64, AIC_2_ = 17444.50, BIC = 20715.12 [df = 653; *n* = 103]. IQ-TREE: WAG + I + G4, Γ(α) = 2.1319, fraction invariant sites 0.0280; quality assessment scores: log-likelihood: −8702.9806 (s.e. 1116.9852), AIC = 18643.96, cAIC = 17217.27, BIC=20133.72.

VKOR nr 327 domain22-148_SsVKOR MSA.fa: TOPALi2: WAG + I + G, Γ(α) = 1.572, ρINV = 0.007; quality assessment scores: *l* = −45092.52, AIC_1_ = 91491.04, AIC_2_ = 72923.13, BIC = 94370.88 [df = 653; *n* = 608]. IQ-TREE: WAG + I + G4, Γ(α) = 1.4495, fraction invariant sites 0.0147; quality assessment scores: log-likelihood: −44837.1457 (s.e. 3044.1722), AIC = 91018.2907, cAIC = 77102.7215, BIC = 93981.9282.

VKOR nr 327 loop36-79_HsVKORC1 MSA.fa: TOPALi2: WAG + I + G, Γ(α) = 2.141, ρINV = 0.019; quality assessment scores: *l* = −8844.32, AIC_1_ = 18994.64, AIC_2_ = 17444.50, BIC = 20715.12 [df = 653; *n* = 103]. IQ-TREE: WAG + I + G4, Γ(α) = 1.3215, fraction invariant sites 0.0281; quality assessment scores: log-likelihood: −8947.5936 (s.e. 1135.6461), AIC = 19133.1873, cAIC=17706.4958, BIC = 20622.0465.

4. Homology Modeling of human VKORC1 and VKORC1L1 targets using the *E. coli* DsbB template PDB structure 2K74 (model 1)

The nearly identical lengths of DsbB (176 aa), VKORC1 (163 aa) and VKORC1L1 (176 aa) suggested to us that indels would not be a major conern in modeling, so we attempted pairwise aligments using ClustalW2 realizing that approximately the first helix pair and loop of DsbB would correspond to the second helix pair and loop, and *vice versa,* of the VKOR proteins.

Figure S1 illustrates the discrete topological transformational operations required to produce the final modeling input sequences for VKORC1 (C1) and VKORC1L1 (L1). Because all three of the essential cut-and-paste operations are located at sequence sites outside of the four-helix core bundle, we were assured that the bundle interhelical spatial relationships would remain undisturbed throughout the modeling process, but that judicious choices of sequence splices at the new topological junctions would be required. The resulting initial models (referred to as Ψ-VKORC1 and Ψ-VKORC1L1) would then accurately represent the portion of the proteins embedded in the lipid bilayer, but require resplicing of the N- and C-termini as well as opening and closing of the two cytoplasmic loops between the yellow/red and blue/green helix pairs, respectively (indicated in the transition from panel E to panel F in Figure S1). However, the connectivity of the large and small ER lumenal loops are preserved between the actual DsbB, C1 and L1 native structures, thus, not requiring any intervention after the initial modeling round.


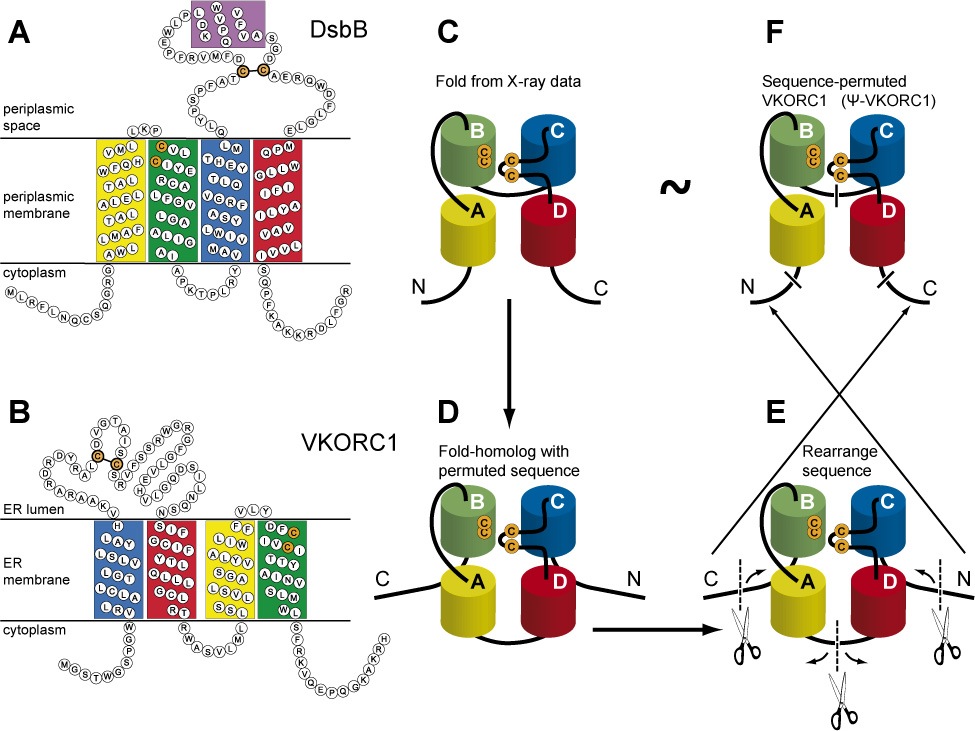


**Figure S1.** Rationale for constructing a template-based model of VKORC1 using sequence/structure alignment to a high-resolution structure of DsbB. Topology and threading of primary sequences for DsbB and VKORC1 are shown in panels A and B, respectively. Sequences thread left-to-right through the four colored transmembrane helices from N-terminus to C-terminus. A schematic of the three-dimensional fold of DsbB originally solved from X-ray diffraction data is shown in panel C with the primary sequence threading through the helices in the order “ABCD”. Panel D shows the homologous four helical bundle fold for VKORC1 with the primary sequence threaded through the structure in the helix order “CDAB”, *i.e.*, circularly permuted with the N-terminus beginning at helix C and threaded in the same direction through the structure, and with the helix bundle oriented in the same view as for DsbB in panel C. Panel E shows the topological cut-and-paste operations that transforms the VKORC1 sequence threading to that of DsbB: effectively, the N- and C-termini are moved from helices C and D, respectively, to helices A and D, while the severed ends of helices B and C are joined together. The resulting sequence-permuted VKORC1 (referred to elsewhere in the manuscript as pseudo-VKORC1, Ψ-VKORC1, or C1db) is shown in panel F and is topologically equivalent to the DsbB fold in panel C.

Following are the contents of the Modeller alignment files showing the L1db and C1db threading alignments relative to the native sequence of the 2K74 PDB atomic coordinate file:

C; Alignment of L1DsbB to DsbB in PIR format

>P1;2K74

structureN:2K74:1:A:183:A:DsbB[CSSC]1-183GHHHHHH-UQ2:E.coliK12::1.00

MLRFLNQASQGRGAWLLMAFTALALELTALWFQHVMLLKPCVLSIYERAALFGVLGAALIGAIAPKTPLRYV

AMVIWLYSAFRGVQLTYEHTMLQLYPSPFATSDFMVRFPEWLPLDKWVPQVFVASGDCAERQWDFLGLEMPQ

WLLGIFIAYLIVAVLVVISQPFKAKKRDLFGRGHHHHHH*

>P1;L1db

sequence:L1db:1::183::pseudo-L1:human::

MAAPVLLRVSAALILMTSSIMSVVGSLYLAYILYFVLKEFCIICIVTYVLNFLLLIINYKRLVYLPRWERVA

RYAVCAAGILLSIYAYHVEREKERDPEHRALCDLGPWVKCSAALASRWGRGFGLLGSIFGKDGVLNQPNSVF

GLIFYILQLLLGMTASAVANEAWKRQLQPKQD-------*

C; Alignment of C1DsbB to DsbB in PIR format

>P1;2K74

structureN:2K74:1:A:183:A:DsbB[CSSC]1-183GHHHHHH-UQ2:E.coliK12::1.00

MLRFLNQASQGRGAWLLMAFTALALELTALWFQHVMLLKPCVLSIYERAALFGVLGAALIGAIAPKTPLRYV

AMVIWLYSAFRGVQLTYEHTMLQLYPSPFATSDFMVRFPEWLPLDKWVPQVFVASGDCAERQWDFLGLEMPQ

WLLGIFIAYLIVAVLVVISQPFKAKKRDLFGRGHHHHHH*

>P1;C1db

sequence:C1db:1::183::pseudo-C1:human::

MGSTWGSPGWASVLMLLSSLVSLAGSVYLAWILFFVLYDFCIVCITTYAINVSLMWLSFRKVQEP----GWV

RLALCLTGLVLSLYALHVKAARARDRDYRALCDVGTAISCSRVFSSRWGRGFGLVEHVLGQDSILNQSNSIF

GCIFYTLQLLLGCLRTRWRKVQEPQGKAKRH--------*

We noted that the organization of the primary sequences for large DsbB and C1/L1 loops differ in the location and spacing of the conserved cystein pairs, with those of DsbB separated by a small amphipathic α-helix in the distal portion of the loop that is apparently anchored down into the membrane allowing each half-loop containing the evenly spaced cysteins to move together or apart during the catalytic cycle [1]. We anticipated that the C1 and L1 loops resulting from first round DsbB template-based modeling would be strongly biased to be spatially constrained to the DsbB loop orientation, whereas recent advances in template-based modeling (TBM) have strived for and, indeed, resulted in models closer to the target protein native state and less restricted to the form of the initial structural template [2,3]. Accordingly, we sought alternative methods for modeling the C1/L1 loops including *de novo* modeling using methods that assemble known structural fragments from the PDB and *ab intio* methods that assemble protein structure based on physical first principles embodied in computational representations of molecular mechanics force fields.

In order to investigate alternative models for the approximate loop region (His28 through Asn80, 53 residues), we used two server-based *de novo* methods, SAM-T08 and I-TASSER, and found I-TASSER returned loop models in compact globular forms for both C1 and L1 loops with substantially buried hydrophobic residues and loop termini at reasonable distance for splicing to the helix bundle. I-TASSER returned 5 model variations ranked by C-scores for which the best ranked models reported TM-scores and RMSD values indicative of structures estimated to have the same fold as the native protein (C1loop: C-score, -1.58, TM-score, 0.52±0.15; RMSD_expect_, 5.8±3.6 Å; L1loop: C-score, -1.50, TM-score, 0.53±0.15; RMSD_expect_, 5.7±3.6 Å). Interestingly, I-TASSER was able to accurately place the secondary structural elements (SSEs) for the DsbB loop when we submitted the loop sequence to the server, but this is not suprising because the DsbB structure data in the PDB was used for progressive fragment assembly and, in fact, I-TASSER accurately reproduced the entire fold of DsbB (helix bundle and loop with accurately placed amphipathic α-helix) – something rarely observed when a membrane protein sequence is submitted to an automated *de novo* modeling server because the algorithms have no way of interpreting protein placement in a native lipid bilayer environment. Subsequently, we performed reorganization of the cytoplasmic loop and N- and C-termini of the helical bundle of the TBMs and spliced the I-TASSER *de novo* modeled ER lumenal loops onto the TBM helix bundles of C1 and L1 using Coot (version 0.6-pre-1) and optimized dihedral angles and bond distances at the splice sites. The initial models were again submitted to Modeller in two subsequent rounds, threading the same sequences through successive modeling round decoys, to produce the final first-round TBMs (C1v3 and L1v3 for human VKORC1 and VKORC1L1, respectively). Following in line with recently reported CASP8 inovations in model quality assessment methods [3], we submitted the atomic coordinate files to the MolProbity server in order to add hydrogen atoms and assess the overall stereochemical quality of the models compared to that of the original DsbB structure used to compute the TBMs (Figure S2, S3). Currently, it is not typically advantageous to refine homology models when detailed experimental data interpretable as intramolecular structural constraints are lacking, especially where the percent identity between target and template sequences is very low [4]. However, despite many clashes between atoms that are too closely spaced for a native structure and the likelyhood that side chain rotamers are not in native configurations, we assessed an overall good quality for the models judged by placement and nature of interhelical contacts and placement of tryptophans, arginines, lysines, large non-polar and small polar residues in appropriate structural regions and side chain orientations relative to the expected positional distribution of these residue types for membrane proteins in a lipid bilayer environment (Figure S4) [5,6]. Notably, the VKORC1 model ER lumenal loop contains a small helical turn spanning residues V66 through V69, correllating well with the independent consensus for a small helix from *ab initio* modelling results (*ab initio* helix consensus from Gly62 though Val69). For the VKORC1L1 TMB, there is a small helical turn at this position, but strictly defined α-helical structure is absent.


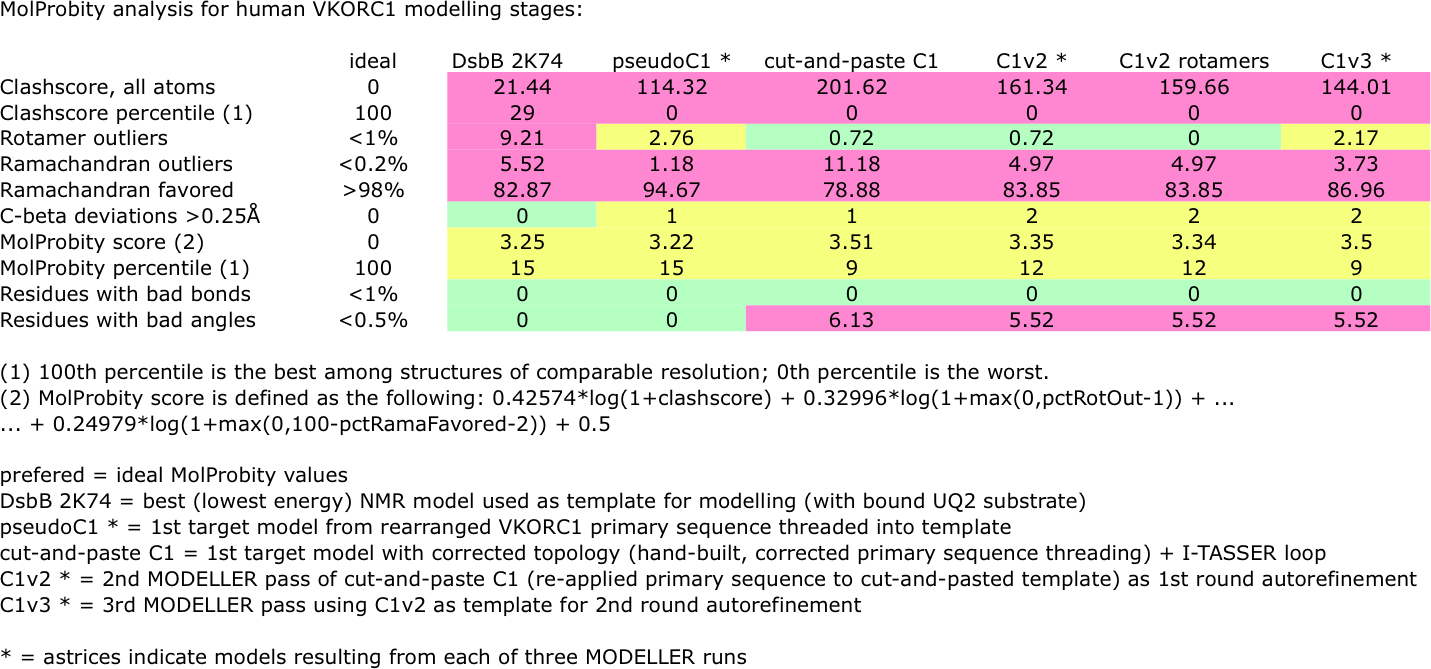


**Figure S2.** Structural validation for human VKORC1 intermediate and final modeling stages.


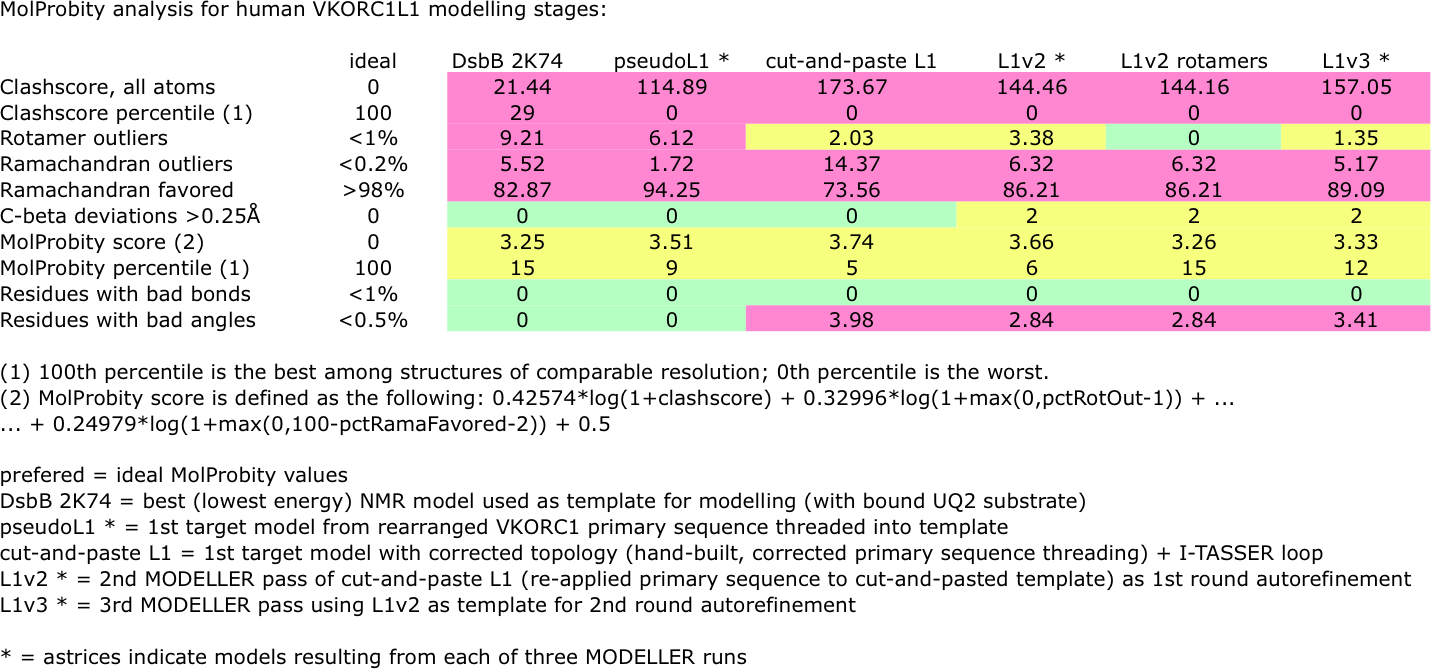


**Figure S3.** Structural validation for human VKORC1L1 intermediate and final modeling stages.


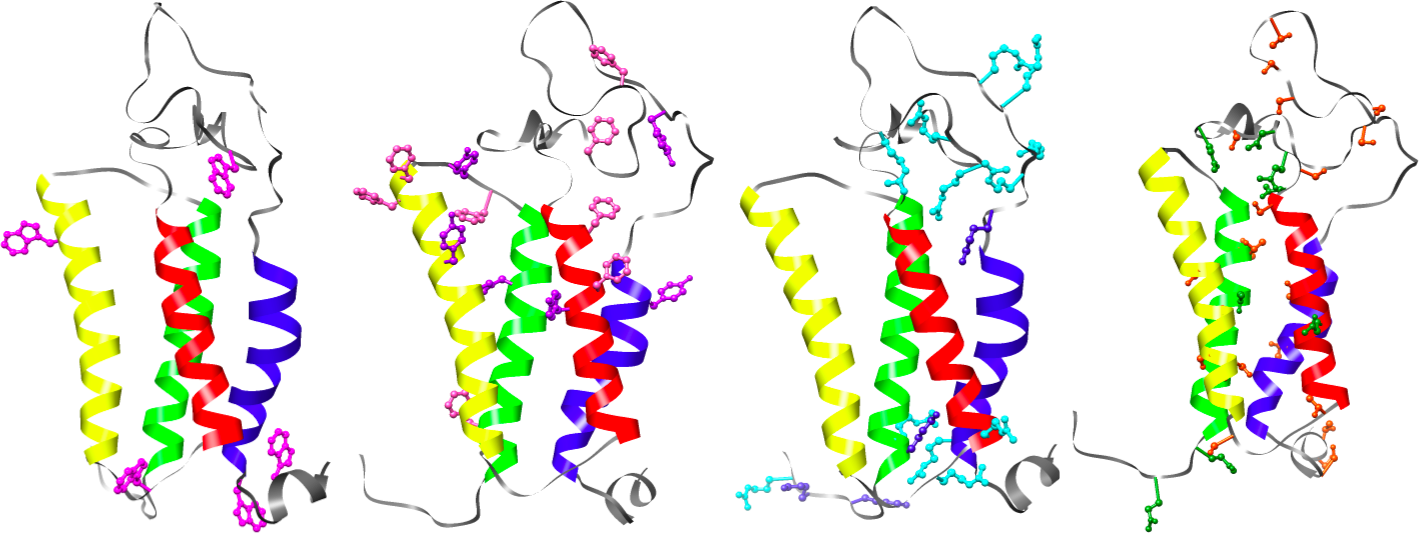


**Figure S4.** Spatial distribution of tryptophans, tyrosines, arginines, lysines, serines, threonines, asparigines and glutamines in human VKORC1 model C1v3 are consistent with placement of the protein in a lipid bilayer. Left-most panel shows tryptophans (magenta sidechains) clustered around the protein periphery in upper and lower rings corresponding to the ER lumenal and cytoplasmic, respectively, aqueous milleau/lipid head group transition interfaces. Second panel from left indicates tyrosines (purple) and phenylalanines (pink) predominantly clustered around the quinone binding pocket in the upper half of the protein. Third panel from left shows arginines (cyan) and lysines (deep blue) predominantly cluster at the cytoplasmic membrane interface in accordance with the "positive-inside" rule of Elofsson & von Heijne [7]. Although there are as many positively charged residues at the ER lumenal side fo the protein, we believe this substantially explains why many transmembrane helix and topology prediction algorithms have trouble consistently predicting a consistent topology for VKORC1 (three or four TMH) as opposed to the uniformly consistent predictions for DsbB and VKORC1L1 (both four TMH). Right-most panel shows the distribution of residues with polar side chains capable of acting as H-bond donors: serines and threonines (orange) predominantly located in the ER lumenal loop and at interhelical interfaces of adjoining TM helices; amide-containing side chains of asparigines and glutamines (dark green) are located predominantly clustered in the C-terminal region of the large ER lumenal loop, the "metazoan stretch" that is absent in all prokaryotic and plant VKOR proteins.

5. Overview of indels for DsbB and VKOR multiple sequence alignments


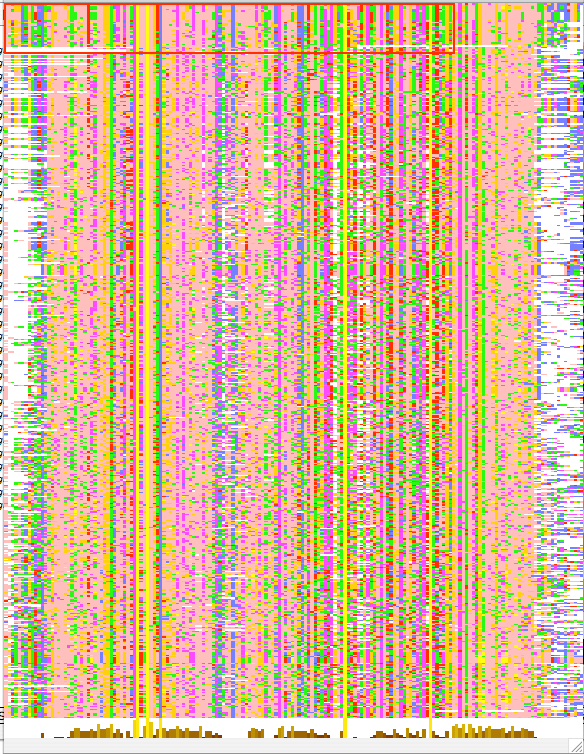


**Figure S5.** DsbB multiple sequence alignment overview. Indels appear as white gaps.

**Figure S6.** VKOR multiple sequence alignment overview. Indels appear as white gaps.
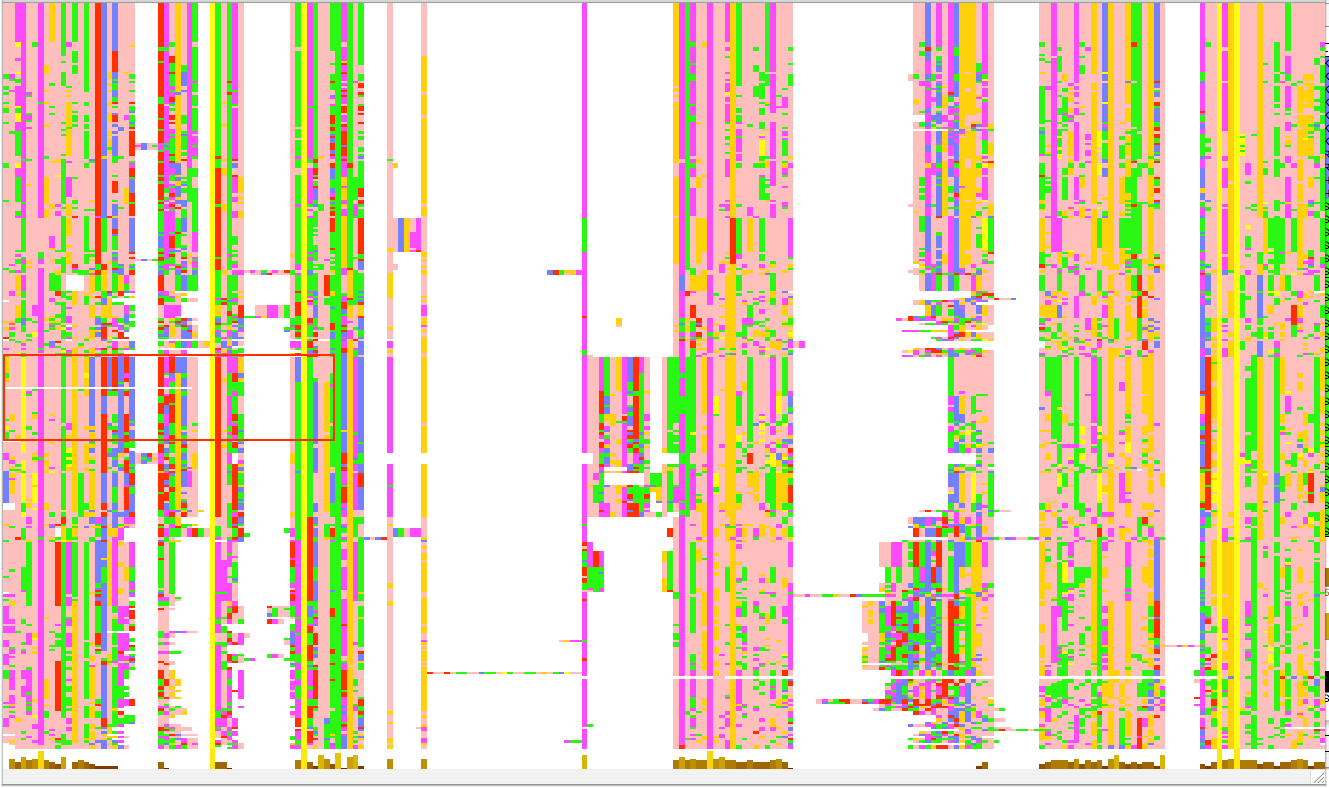


6. Domain analysis for VKOR family sequences


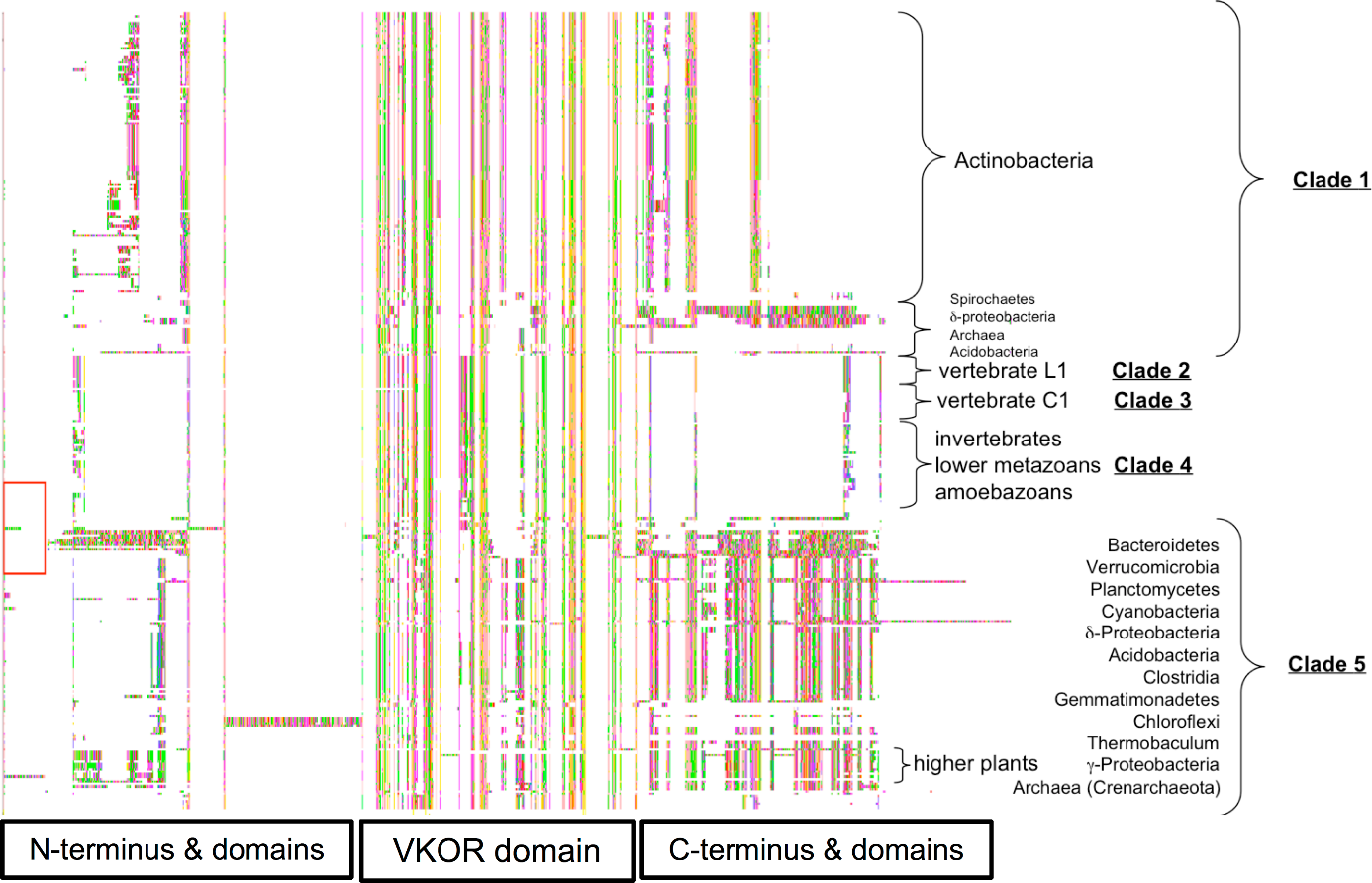


**Figure S7.** Full-length VKOR family sequences ordered according to the unrooted reconstructed tree shown in Figure Figure3 of the main article. Indels appear as white gaps.

References

1. Inaba, K.; Murakami, S.; Nakagawa, A.; Iida, H.; Kinjo, M.; Ito, K.; Suzuki, M. Dynamic nature of disulphide bond formation catalysts revealed by crystal structures of DsbB. *Embo. J.* **2009**, *28*, 779–791.
2. Kryshtafovych, A.; Fidelis, K.; Moult, J. CASP8 results in context of previous experiments. *Proteins* **2009**, *5*, 5.
3. Keedy, D. A.; Williams, C. J.; Headd, J. J.; Arendall, W. B., 3rd; Chen, V. B.; Kapral, G. J.; Gillespie, R. A.; Block, J. N.; Zemla, A.; Richardson, D. C.; Richardson, J. S. The other 90% of the protein: Assessment beyond the Calphas for CASP8 template-based and high-accuracy models. *Proteins* **2009**, *20*, 20.
4. Punta, M.; Forrest, L. R.; Bigelow, H.; Kernytsky, A.; Liu, J.; Rost, B. Membrane protein prediction methods. *Methods* **2007**, *41*, 460–474.
5. Ulmschneider, M. B.; Sansom, M. S.; Di Nola, A. Properties of integral membrane protein structures: derivation of an implicit membrane potential. *Proteins* **2005**, *59*, 252–265.
6. Liang, J.; Adamian, L.; Jackups, R., Jr. The membrane-water interface region of membrane proteins: structural bias and the anti-snorkeling effect. *Trends Biochem Sci* **2005**, *30*, 355–357.
7. Elofsson, A.; von Heijne, G. Membrane protein structure: prediction versus reality. *Annu. Rev. Biochem.* **2007**, *76*, 125–140.

© 2015 by the authors; licensee MDPI, Basel, Switzerland. This article is an open access article distributed under the terms and conditions of the Creative Commons Attribution license (http://creativecommons.org/licenses/by/4.0/).
